# The influence of early environment and micronutrient availability on developmental epigenetic programming: lessons from the placenta

**DOI:** 10.3389/fcell.2023.1212199

**Published:** 2023-07-06

**Authors:** Rebecca Sainty, Matt J. Silver, Andrew M. Prentice, David Monk

**Affiliations:** ^1^ Biomedical Research Centre, School of Biological Sciences, University of East Anglia, Norwich, United Kingdom; ^2^ Medical Research Council Unit The Gambia at London School of Hygiene and Tropical Medicine, London, United Kingdom; ^3^ Medical Research Council Unit The Gambia at London School of Hygiene and Tropical Medicine, Banjul, Gambia

**Keywords:** epigentics, DNA methylation, placenta, imprinting, metastable epialles, DOHaD

## Abstract

DNA methylation is the most commonly studied epigenetic mark in humans, as it is well recognised as a stable, heritable mark that can affect genome function and influence gene expression. Somatic DNA methylation patterns that can persist throughout life are established shortly after fertilisation when the majority of epigenetic marks, including DNA methylation, are erased from the pre-implantation embryo. Therefore, the period around conception is potentially critical for influencing DNA methylation, including methylation at imprinted alleles and metastable epialleles (MEs), loci where methylation varies between individuals but is correlated across tissues. Exposures before and during conception can affect pregnancy outcomes and health throughout life. Retrospective studies of the survivors of famines, such as those exposed to the Dutch Hunger Winter of 1944-45, have linked exposures around conception to later disease outcomes, some of which correlate with DNA methylation changes at certain genes. Animal models have shown more directly that DNA methylation can be affected by dietary supplements that act as cofactors in one-carbon metabolism, and in humans, methylation at birth has been associated with peri-conceptional micronutrient supplementation. However, directly showing a role of micronutrients in shaping the epigenome has proven difficult. Recently, the placenta, a tissue with a unique hypomethylated methylome, has been shown to possess great inter-individual variability, which we highlight as a promising target tissue for studying MEs and mixed environmental exposures. The placenta has a critical role shaping the health of the fetus. Placenta-associated pregnancy complications, such as preeclampsia and intrauterine growth restriction, are all associated with aberrant patterns of DNA methylation and expression which are only now being linked to disease risk later in life.

## 1 Introduction

According to the Developmental Origins of Health and Disease hypothesis, environmental exposures in early life affects later life risk. Part of this connection may come through DNA methylation, patterns of which are known to change under different conditions. The placenta is important for mediating the connection between mother and fetus, both able to respond to the environment itself and controlling the environment of the fetus. Many pregnancy complications are linked to placenta function and birth outcomes can have a large effect on later disease risk. This review will summarise current knowledge on the effect of early environmental exposure on later disease risk, especially where this may be mediated by DNA methylation. We will highlight the unique nature of the placenta epigenome and its potential as a connection between environment and health.

### 1.1 Epigenetic processes in regulating transcription

Epigenetic marks are heritable DNA modifications that can influence gene expression without changing the DNA sequence. These include chemical modifications of DNA bases, post-translational histone modifications and chromatin structure, and their configuration can be affected by a variety of environmental exposures.

### 1.2 Histone modifications

Histones wrapped with DNA form the nucleosome, that can alter gene accessibility by forming transcriptionally inactive heterochromatin or transcriptionally active euchromatin (Li et al., 2007). Expression can be controlled by reversible post-translational modifications on histone amino acid tails, with complex cross-talk between modifications (Kouzarides, 2007). For example, lysine 9 or 27 acetylation on histone 3 (H3K9ac or H3K27ac) weakens DNA-histone interactions, and so opens chromatin to facilitate transcription, whereas trimethylation of the same lysines is associated with heterochromatin formation (Quina et al., 2006).

### 1.3 Cytosine methylation in the human genome

5-methylcytosine (5 mC) is well established as a stable, heritable DNA methylation mark that affects gene expression and genome stability. It is created by the addition of a methyl group to the 5-carbon atom of the cytosine ring (Moore et al., 2013). This modification is important for many processes, including tissue-specific gene regulation, imprinting and X-chromosome inactivation, working in combination with histone modifications (Mikkelsen et al., 2007; Moore et al., 2013). In promoters and intergenic regions, it is generally associated with transcriptional silencing through recruiting gene suppressor proteins, promoting heterochromatin and disrupting transcription factor binding (McMahon et al., 2017; Kaluscha et al., 2022). In bodies of highly expressed genes, high levels of 5 mC stop promiscuous transcription initiation (Neri et al., 2017).

Most 5 mC is found on CpG dinucleotides (a cytosine base next to a guanine), up to 80% of which are methylated in mammalian genomes (Lister et al., 2009). These CpG dinucleotides are often clustered into CpG islands, which are associated with 70% of known gene promoters, where methylation can silence gene expression (Mohn et al., 2008; Illingworth et al., 2010). They are also enriched in repetitive elements, satellite DNA and transposable elements to help maintain genome stability (Vera et al., 2008; Lister et al., 2009; Si et al., 2009).

In humans, 5 mC is created and maintained by DNA methyltransferases (DNMTs) using S-adenyl methionine (SAM) as a methyl donor. DNMTs include: DNMT3a and 3b for *de novo* methylation in embryos and germ cells, with the non-catalytic DNMT3L as a cofactor (Bourc’his and Bestor, 2004; Kaneda et al., 2004), and DNMT1 for maintenance during DNA replication and repair (Hermann et al., 2004; Mortusewicz et al., 2005). Methylation can be removed passively through replication without methylation maintenance, or actively, through several intermediates catalysed by Ten-Eleven-Translocation (TET) proteins (Hackett et al., 2013). One of these intermediates, 5-hydroxymethylcytosine (5hmC), has been proposed to also play a regulatory role as its distribution shows strand and sequence bias. 5hmC is enriched in regulatory elements and promoters in embryonic stem cells and occasionally in placenta (Hernandez Mora et al., 2018), as well as in actively transcribed genes in neuronal cells (Mellén et al., 2012; Yu et al., 2012).

### 1.4 Non-cytosine methylation

Another suggested regulatory modification is methylation to the adenine base (m6dA), which is a known epigenetic mark in bacteria, protists (Wion and Casadesús, 2006), and eukaryotes such as *C. elegans* (Greer et al., 2015). In humans, m6dA is a common RNA modification but is also detected on DNA, where it has been associated with actively transcribed genes and risk of tumorigenesis (Xiao et al., 2018). However, suggestions for a human DNA adenine methylase or demethylase have failed to be replicated (Xie et al., 2018; Musheev et al., 2020) and other studies suggest DNA m6dA in humans arises purely through nucleotide salvage from RNA degradation (Liu et al., 2020; Musheev et al., 2020).

### 1.5 Methyl donors: the one-carbon metabolism pathway

**FIGURE 1:**
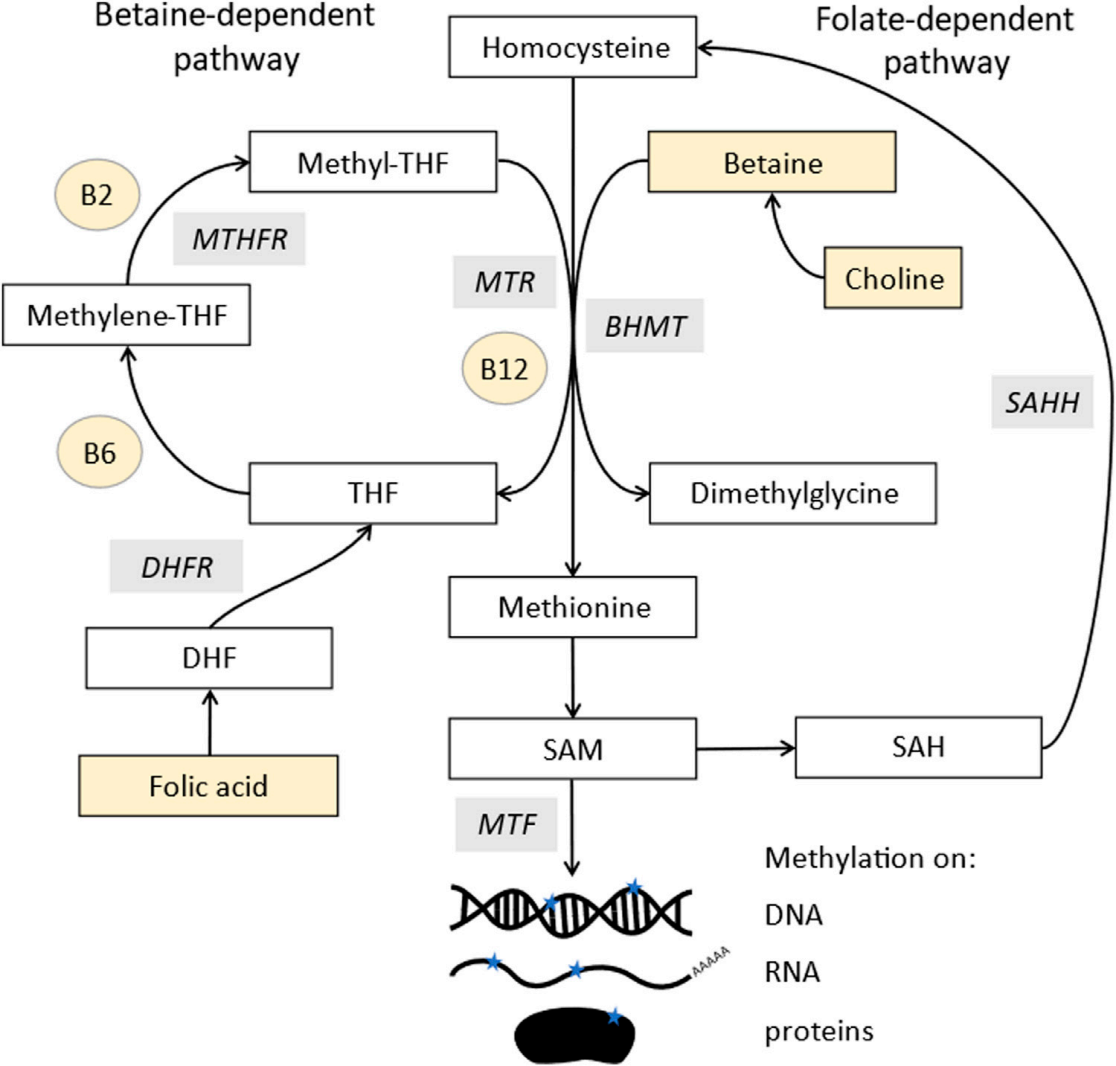
Methylation pathway. Homocysteine (top middle) is converted into methionine by two pathways: methionine synthase (MTR), which uses vitamin B12 as a cofactor and acquires a methyl group from the conversion of 5-methyltetrahydrofolate (methyl-THF) into tetrahydrofolate (THF). MethyL-THF is acquired from dietary folates which is converted from dihydrofolate (DHF) by dihydrofolate reductase (DHFR) and 5,10- methylenetetrahydrofolate (5,10MTHF) by methylene tetrahydrofolate reductase (MTHFR). Methionine can also acquire a methyl group from betaine via a reaction involving betaine homocysteine methyl-transferases (BHMT). Methionine is further converted to s-adenosylmethionine (SAM), the major methyl donor for all methyl transferases (MTF), which add methyl groups to DNA, RNA, lipids, and proteins. SAM is recycled via s-adenosylhomocysteine (SAH) which is converted to homocysteine in a reversible reaction by s-adenosylhomocysteine hydrolase (SAHH). Components highlighted in yellow are derived from the diet.

The synthesis of both post-translational protein methylation, including histones, and DNA methylation is dependent on one-carbon metabolism, along with many other cellular processes (Ducker and Rabinowitz, 2017). The methyl group is donated from S-adenosylmethionine (SAM), leaving S-adenosyl homocysteine (SAH) (Figure 1). Many components of this pathway must come from the diet, both methyl donors, including folate, choline and betaine, and cofactors, including vitamins B-2, B-6, and B-12 (James et al., 2018). Studies in animal models demonstrate that supplementing these nutrients can alter DNA methylation patterns (Waterland and Jirtle, 2003; Sinclair et al., 2007).

SAM is derived from methionine, which comes from methylated homocysteine. There are two enzymes that can make methionine, most commonly methionine synthase, which requires vitamin B12 as a cofactor. It uses 5-methyltetrahydrofolate as a methyl donor, whose synthesis is folate dependent and also links into purine biosynthesis. The second enzyme, betaine-homocysteine methyltransferases (BHMT), has two isoforms, of which one is expressed only in the liver and kidneys, with the other (BHMT2) expressed more widely (Pajares and Pérez-Sala, 2006). This uses betaine as a methyl donor, which comes either from choline or direct dietary supplementation.

The donation of the methyl group from SAM is catalysed by methyl transferases, which are inhibited by SAH. Therefore, methylation stops if the process of converting SAH back to homocysteine and methionine and SAM is inhibited. Ways to measure methylation potential therefore include the SAM:SAH ratio as well as levels of the pathway components (Mason, 2003).

### 1.6 Methylation life cycle

DNA methylation can change dynamically throughout life but there are many loci where it remains consistent. For these loci, there are two major reprogramming events to consider. The first is in the primordial germ cells that give rise to the sperm and oocyte, where DNA is passively demethylated during initial formation of the germ cells to remove any previous marks (Hackett et al., 2013; Guo et al., 2015). The DNA can then be remethylated to establish parent-of-origin (PoO) specific methylation, which according to studies in mice occurs before birth in sperm but not until maturation for oocytes (Hiura et al., 2006; Guo et al., 2014; 2015; Gahurova et al., 2017).

The next major reprogramming stage occurs after fertilisation, where a wave of demethylation before the blastocyst stage results in the lowest developmental level of genome methylation (Weaver et al., 2009). Parental DNA from sperm begins almost entirely methylated, except in CpG islands, but at this stage most of its methylation is actively removed (Guo et al., 2014; Shen et al., 2014; Smith et al., 2014). In contrast, maternal, oocyte-derived DNA is methylated mostly at active gene bodies and maintains more methylation (Smallwood et al., 2011). It is demethylated passively by dilution through replication (Hirasawa et al., 2008; Shen et al., 2014).

Differential maintenance of methylation at this stage has the potential to create changes in gene expression. This can create metastable epialleles, loci with variable methylation between, but not within, individuals. Regions that maintain PoO specific methylation through this process create germline differentially methylated regions (gDMRs), with the potential to be imprinted.

Where differential methylation is maintained, alleles are protected against demethylation by ZFP57 and ZFP445, which targets DNMT1 (Li et al., 2008; Quenneville et al., 2011; Takahashi et al., 2019). The opposite alleles are kept unmethylated by many different factors, either acting together or on different genes, including CFP1, SP1 or CTCF (CCTC-binding factor) (Macleod et al., 1994; Fedoriw et al., 2004).

### 1.7 Imprinting

**FIGURE 2:**
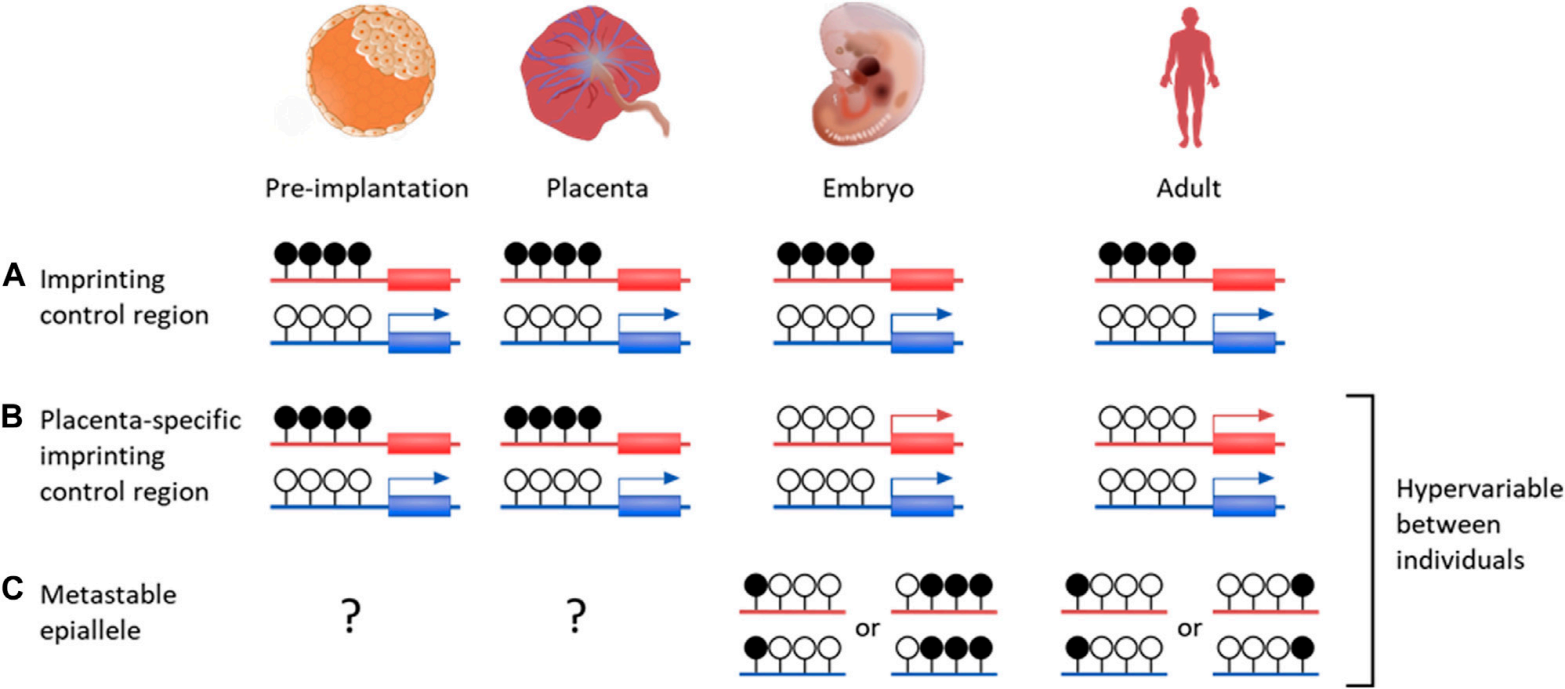
DNA methylation profiles of various genetic features that are important for placenta development and growth. Canonical genomic imprinting is associated with regions of allelic methylation inherited from one gamete. **(A)** Oocyte-derived methylation is faithfully maintained throughout development on the maternal allele, resulting in paternal expression. **(B)** An example of a germline-derived, placenta-specific imprint. During pre-implantation development these regions are indistinguishable from canonical imprinted DMRs. Upon implantation, embryonic tissues become demethylated through to adulthood, while allelic methylation is maintained solely in the placenta. **(C)** While establishment of metastable epialleles during early development is currently unknown, these intervals are particularly vulnerable to environmental influences. Once systematically methylated, they are stable over time, despite showing inter-individual variability. Blue genes are paternally expression, red genes are maternally expressed. Black filled lollipops are methylated CpG intervals, while unfilled are unmethylated.

Imprinted genes are found in therian (eutherian and marsupial) mammals and are defined by differential expression between maternal and paternal alleles, which can be specific to developmental stage, tissue or isoform (Renfree et al., 2013). Incorrect imprinting can have long term effects, such as imprinting diseases (Peters, 2014; Monk et al., 2019). Imprinting is generally a result of allele-specific repression from gDMRs, with most imprinted genes found within regions containing an imprinting control region (ICR) (Figure 2) (Spahn and Barlow, 2003; Schulz et al., 2010). As well as directly controlling expression, imprinted DMRs are often associated with repressive histone modifications, such as H3K9me3 on methylated alleles (Monk et al., 2006; Court et al., 2014).

There are over 120 confirmed imprinted genes in humans, of which most are associated with maternally methylated regions (Okae et al., 2014; Humanimprints.net; Geneimprint, 2023). Some gDMRs maintain allelic methylation throughout life but the majority are transient, surviving the pre-implantation demethylation then becoming entirely methylated or unmethylated after implantation (Proudhon et al., 2012). It is possible these have a function as ICRs in the pre-implantation embryo, or in the placenta, where many transient gDMRs are maintained (Figure 2) (Sanchez-Delgado et al., 2016).

Examples of non-canonical imprinting are so far restricted to mice. In these cases, imprinted genes have no detectable methylation differences between parental gametes. Instead, they have the histone modification H3K27me3 in the oocyte, which later leads to maternal allele methylation in extra-embryonic tissues (including placenta) (Inoue et al., 2017).

### 1.8 Metastable epialleles

MEs are loci with variable methylation between individuals, but consistent cross-tissue methylation within individuals, suggesting the methylation is established early in development before separation of the germ layers. They are often associated with transposable elements, which evolve fast and often give rise to new cis-regulatory DNA elements or even new exons and genes (Imbeault et al., 2017; Bourque et al., 2018).

The Agouti viable yellow (*A^vy^*) mouse allele is a well characterised example of a mammalian metastable epiallele associated with a transposable element, which causes a yellow coat colour when expressed (Dickies, 1962; Duhl et al., 1994). This allele was created by insertion of an Intracisternal A-particle (IAPs), a Class II endogenous retrovirus (ERV) with protein-coding sequences between long terminal repeats (LTRs) (Cole et al., 1981). The insertion created a cryptic promoter in the LTR which drives expression when unmethylated and crucially the epigenetic states, including DNA methylation and histone profiles, are variable between individuals (Duhl et al., 1994; Morgan et al., 1999; Kazachenka et al., 2018). The phenotype is heritable, with maternal inheritance causing an incomplete shift in yellow coats in the litter (Morgan et al., 1999). The pattern in phenotype can also be shifted by environment, for example, a preconception diet rich in methyl donors and cofactors *in utero* leads to increased methylation and fewer offspring with yellow coats (Wolff et al., 1998; Cooney et al., 2002; Waterland and Jirtle, 2003; Cropley et al., 2006).

Subsequent studies have found other variably methylation IAPs, which vary between but not within individuals (Kazachenka et al., 2018). Most are not heritable and many are not changed by environmental exposures like bisphenol A or methyl donor supplementation (Bertozzi et al., 2021). Sites also seem to function independently, as methylation is generally not correlated across different sites within individuals but is found at consistent levels across populations at each site (Kazachenka et al., 2018).

## 2 Developmental origins of health and disease

The Developmental Origins of Health and Disease (DOHaD) hypothesis states that environmental exposures in early life affect the risk of adverse health outcomes later in life (Barker, 1990). Epidemiological studies have shown many associations between early environment and later disease outcomes in humans.

There are several possible mechanisms that could link early environmental exposures to lifelong health of which DNA methylation changes, causing gene expression changes, are a leading candidate (Fleming et al., 2018). When investigating these cases, the time period around conception may be a critical window because, as previously discussed, shortly after fertilisation patterns of DNA methylation are established that can persist throughout life.

### 2.1 Early life exposures and health

Adverse birth outcomes can be caused by many different factors, including a wide variety of environmental exposures. Both maternal obesity and preconception physical activity are associated with risk of preeclampsia (Aune et al., 2014; Marchi et al., 2015; Poston et al., 2016). Alcohol and caffeine intake have been linked to reduced birthweight (Konje and Cade, 2008; Popova et al., 2021), and tobacco smoke exposure to worse birth outcomes, including reduced fetal growth (Peterson and Hecht, 2017). These adverse birth outcomes can subsequently be linked to later life disease, as discussed previously with placenta-related outcomes.

Important exposures often occur over long time periods in humans, so the critical windows of sensitivity are easier to separate in animal models. These studies show short term effects during pregnancy, such as placental growth being affected by a preconception zinc deficiency in rodents (Tian et al., 2014). Longer term effects include increased risks of offspring hypertension and adiposity from a preimplantation low protein diet in rodents (Kwong et al., 2000; Watkins et al., 2008a; Watkins et al., 2008b). These models suggest the blastocyst is capable of sensing nutrient status and altering the phenotype of placental development (Eckert et al., 2012; Watkins et al., 2015).

In humans, famines occurring over well-defined dates allow the study of exposures in specific developmental windows. One well-studied example is the Dutch Hunger Winter, a severe famine in 1944-45. Prenatal exposure to this famine increased the risk of diabetes, schizophrenia and other diseases in later life (Lumey et al., 2011).

Many examples of studies on the effect of early life nutrition and health outcomes are from a Sub-Saharan population in rural Gambia that experiences two distinct seasons with very different environmental, especially nutritional, exposures. In the rainy/hungry season, food availability is lower and energy status is poorer than in the dry/harvest season (Prentice et al., 1981). Babies born in the rainy season are more likely to have intrauterine growth restriction (Ceesay et al., 1997). They are also 10 times more likely to die prematurely, which may be linked to infection (Moore et al., 1997; 1999), as babies born in the hungry season have altered T cell immunity, with lower CD3^+^ and CD4^+^ lymphocyte counts (Ngom et al., 2011).

Micronutrition supplementation studies can test the association between nutritional environment and health more directly, for example, folic acid is known to reduce neural tube defects by up to 70% when taken in the months before and after conception and may also reduce the risk of small for gestational age births (Mastroiacovo and Leoncini, 2011; Hodgetts et al., 2015; Gao et al., 2016). The UK based UPBEAT trial used an intervention during pregnancy that reduced processed and snack food consumption and found a decrease in infant adiposity at 6 months of age (Patel et al., 2017).

An alternative approach to isolating early life environment is the study of assisted reproductive technologies (ART), such as *in-vitro* fertilisation, which provide a different environment only around conception. These show increased risk of high blood pressure in children and altered heart shape and chamber size in infants (Ceelen et al., 2008; Valenzuela-Alcaraz et al., 2013).

### 2.2 Early life environment and methylation

As discussed, methylation could be a mechanism for the link between early environmental exposures and health. In humans, ART has been linked to an increased risk of imprinting disorders (Lazaraviciute et al., 2014) and changes in methylation and histone modifications at imprinted genes (Choux et al., 2020). However, another study found no variability in 25 imprinted DMRs and allelic expression in placenta or cord blood in spontaneously conceived and assisted pregnancies (Camprubí et al., 2013).

Many environmental factors are known to affect methylation, for example, there are changes in methylation at repetitive regions with fetal bisphenol A exposure (Faulk et al., 2016) and changes in methylation at 12 CpGs in blood and 12 in buccal epithelial cells with prenatal exposure to phthalates (England-Mason et al., 2022). Aflatoxin B1 exposure *in utero* in The Gambia was associated with differential methylation at 71 CpG sites in infant white blood cells, including in growth factor and immune-related genes (Hernandez-Vargas et al., 2015).

### 2.3 Early life nutrition and methylation

Nutrition exposure may be key to investigating methylation variability, due to the direct links of micronutrients into the 1C pathway that provides methyl groups required for DNA methylation. Again, animal studies can provide more detailed exposure timing and insight into molecular mechanisms. In mouse studies of periconceptional maternal obesity, fetal growth rate is directly affected by alterations in methylation at the ribosomal DNA promoter which controls the levels of ribosomal RNA (Denisenko et al., 2016; Holland et al., 2016).

In humans, data from the Dutch Hunger Winter shows adults whose mothers were exposed to the famine only before and during the periconceptional period have modestly decreased DNA methylation in the *IGF2* gene (Heijmans et al., 2008). Further studies have shown exposure to this famine in early gestation affects methylation in many regions, mostly annotated as regulatory and with nearby genes expressed in early developmental phases (Tobi et al., 2014).

Identification of MEs in humans is complex, but advances in molecular techniques have suggested their existence in the human genome and identified putative links with environment and disease. They are a useful tool when exploring the link between environment, methylation and health because they are established very early in development and are hypervariable. They can also be studied in easily available samples, such as fetal blood obtained from the umbilical cord.

**FIGURE 3:**
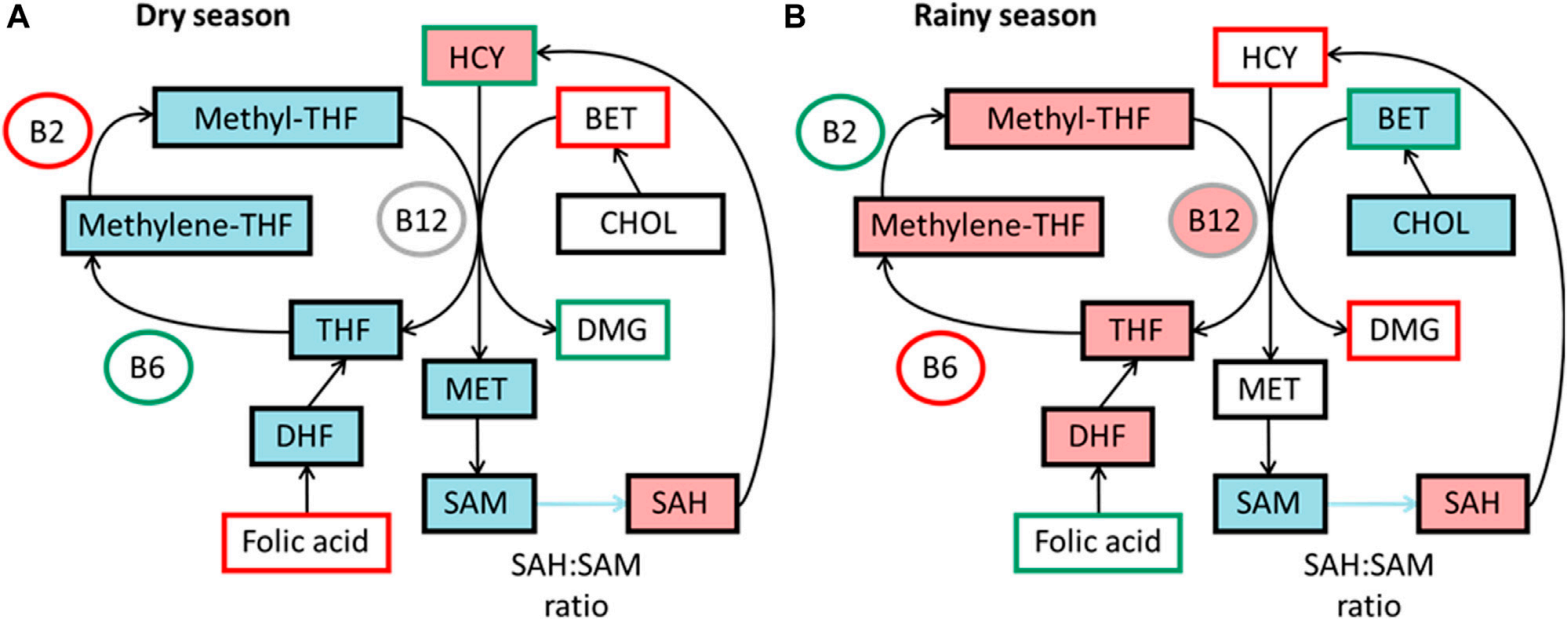
Simplified summaries of metabolic pathways highlighting seasonal differences reported from studies in The Gambia. Comparative levels between seasons in diet as measured by blood biomarkers and components predicting DNA methylation in each season, data from James et al. (2019) **(A)** Components outlined in green are higher in dry season and components outlined in red are lower. Components highlighted in blue are positive predictors of DNA methylation and pink negative predictors in dry season. **(B)** Components outlined in green are higher in rainy season and components outlined in red are lower. Components highlighted in blue are positive predictors of DNA methylation and pink negative predictors in rainy season. BET, betaine; B12, vitamin B-12; B2, vitamin B-2; B6, vitamin B6; CHOL, choline; DHF, dihydrofolate; DMG, dimethyl glycine; HCY, homocysteine; MET, methionine; methylene-THF, N5,10-methylene tetrahydrofolate; methyl-THF, N5-methyl tetrahydrofolate; SAH, S-adenosyl homocysteine; SAM, S-adenosyl methionine; THF, tetrahydrofolate.

The levels of micronutrients essential for the 1C cycle in diet can affect methylation levels (Waterland and Jirtle, 2003; Sinclair et al., 2007). In The Gambia, micronutrients related to the 1C cycle vary across the seasons, as measured by maternal blood biomarkers (Dominguez-Salas et al., 2013). Studies have found higher concentration of methyl donors in the rainy season, potentially giving a higher methylation potential (Figure 3).

Non-imprinted DMRs in cord blood, placental tissue or peripheral blood from Gambian infants have also been associated with birthweight, and 25 DMRs in infant blood at 12 months are associated with length for age (Quilter et al., 2021). A meta-analysis found DNA methylation in neonatal blood has been linked with many pregnancy exposures and is associated with birthweight through childhood, but not adulthood. There is also an overlap between CpGs related to birthweight and intrauterine exposures (Küpers et al., 2019).

These changes were first associated with significant changes in methylation at seven infant MEs, with increased levels of homocysteine and B6 and decreased levels of B2 correlating with higher methylation (Dominguez-Salas et al., 2014). A model found different biomarkers predicted methylation between seasons, with vitamin B-2 and methionine as positive predictors in the dry season, and choline and vitamin B-6 as positive and folate and vitamin B-12 as negative predictors in the rainy season (Figure 3) (James et al., 2019). The different seasonal effects suggest a switch between the betaine dependent pathway in the rainy season to the folate dependent pathway in the dry season.

In further studies, children conceived in the Gambian rainy season showed consistently increased methylation at multiple MEs (Waterland et al., 2010; Khulan et al., 2012; Silver et al., 2022). Methylation changes extend beyond MEs; Silver et al. (Silver et al., 2022) identified 259 CpGs that showed a robust signature of differential methylation between seasons of conception (SoC-CpGs) across two independent cohorts. In one of these cohorts methylation measured in blood in early and mid-childhood showed that the seasonality effect was attenuated in the older cohort.

Many SoC-CpGs overlapped with MEs identified in previous studies and many have been associated with sex (Kessler et al., 2018). They were also enriched for CpGs previously identified to be methylated on PoO specific alleles (Zink et al., 2018), which were also enriched for placenta-specific oocyte gDMRs (Sanchez-Delgado et al., 2016).

One example of a non-coding RNA gene responsive to SoC is *VTRNA2-1*, which is also a putative ME (Silver et al., 2015). This tumour suppressor gene has been linked to cancer and there is evidence that it is maternally imprinted (Romanelli et al., 2014; Silver et al., 2015).

The EMPHASIS (Epigenetic Mechanisms linking Preconceptional nutrition and Health Assessed in India and sub-Saharan Africa) study conducted two randomised controlled trials that used different micronutrient interventions taken preconception and during pregnancy. Six differentially methylated CpGs were found in Gambian children, of which four were around the endothelial cell-specific molecule 1 (*ESM1*) gene (Saffari et al., 2020). Another randomised controlled trial found sex-specific epigenetic changes in Gambian cord and infant blood from periconceptional micronutrient supplementation (Khulan et al., 2012). In these children, girls had reduced methylation at *IGFR2* and boys at *MEG3/GTL2*, but these differences disappeared by 9 months (Cooper et al., 2012).

One of the genes with increased methylation associated with periconceptional micronutrient supplementation and conception in the Gambia rainy seasons is *PAX8* (paired-box 8) (Waterland et al., 2010; Silver et al., 2015; Saffari et al., 2020). It is a master regulator of thyroid gene expression, and its methylation has been associated with thyroid function in Gambian children (Candler et al., 2021). Another example is *POMC*, where methylation has been separately shown to reduce *POMC* transcription and is associated with obesity in children (Kuehnen et al., 2012; Kühnen et al., 2016).

## 3 Human placenta

### 3.1 Placenta and disease

The placenta is a transient organ that acts as a physical connector and barrier between maternal and fetal blood, mediating nutrient and gas exchange, and waste removal between the embryo and the mother. It also functions as an endocrine organ, producing hormones including human chorionic gonadotropin (hCG) (Lopata et al., 1997). Placenta development and structure is highly species-specific; the human placenta displays deep trophoblast invasion which is unique to great apes (Imakawa et al., 2015). Correct functioning is vital for appropriate fetal development, for example, increased glucose transfer in gestational diabetes increases fetal adiposity and macrosomia, which subsequently is associated with disease in later life (Pettitt et al., 1993; Tint et al., 2020).

Many pregnancy complications are linked with placental development defects, including preeclampsia, fetal growth restriction, recurrent miscarriage and still-birth (Brosens et al., 2011). These conditions contribute to a high proportion of maternal and neonatal morbidity and mortality, especially in sub-Saharan Africa (Graham et al., 2016).

Intrauterine growth retardation (IUGR) affects 10%–15% pregnancies and is a subset of small for gestational age, which is defined as birth weight below the 10th percentile (Harkness and Mari, 2004). Many IUGR cases are caused by placental insufficiency. In these cases, there is deficient remodeling of uterine spiral arteries that supply the placenta creating a lower villous volume and surface area for maternal-fetal exchange, and dysregulation of many genes (Burton and Jauniaux, 2018). Babies with IUGR have a high risk of hypertension, type 2 diabetes and heart disease later in life (Curhan et al., 1996; Rich-Edwards et al., 1997).

Preeclampsia affects 3%–5% of pregnancies in the developed world and forms part of the hypertensive disorders that cause 12% of maternal deaths during and after pregnancy (WHO, 2005). It is characterised by hypertension and proteinuria in the second half of pregnancy (Roberts and Cooper, 2001) and is also associated with defective uterine spiral artery remodeling. Its effects range from mild to multiorgan failure and it is caused by low perfusion in the placenta (Brosens et al., 2011).

Overall placenta morphology has also been associated with later disease risk. For example, in the Helsinki Birth Cohort that experienced prenatal famine exposure, placenta thickness has been associated with risk of sudden cardiac death (Barker et al., 2012). Another study linked longer and more oval placentas in this cohort with colorectal cancer (Barker et al., 2013).

### 3.2 The unique state of the placenta epigenome

The placenta is hypomethylated overall, with around 3% cytosine bases methylated compared to 4% in somatic tissue (Fuke et al., 2004). Global placenta methylation increases from 2.8% in first trimester to 3.1% in term placentas, unlike global methylation in somatic tissues which decreases with age (Fuke et al., 2004). The location of methylation also changes with gestational age, suggesting that genes act at specific stages (Novakovic et al., 2011; Camprubí et al., 2013). Methylation changes with gestational age are accompanied by a change in gene expression levels in 25% of placenta expressed genes (Sitras et al., 2012; Uusküla et al., 2012). Most imprinted genes show reduced expression with advancing gestational age independent of allelic methylation (Monteagudo-Sánchez et al., 2019; Pilvar et al., 2019).

The hypomethylation in placenta is not confined to specific genomic features but does include repetitive elements, including transposable elements, as well as large (>100 kb), partially methylated domains (PMDs) that are largely stable throughout gestation (Schroeder et al., 2013; Schroeder and Lasalle, 2013). Repetitive elements are highly methylated in somatic tissue but variably methylated in placenta and highly species specific, which may allow them to act as drivers for placenta evolution. There are multiple placenta-specific promotors derived from TEs, which includes endogenous retroviruses, including for the *CYP19A1*, *NOS3* and *PTN* genes (Dunn-Fletcher et al., 2018). ERV families have been identified with regulatory potential that are close to trophoblast-specific expressed genes (Sun et al., 2021; Frost et al., 2023). In human trophoblast stem cells, genetic editing showed these elements act as enhancers (Frost et al., 2023).

*HERV-W* is a co-opted human ERV envelope gene which illustrates how these elements can exhibit placenta-specific function. One of its copies produces Syncitin-1, which is important for syncytiotrophoblast fusion, a multinucleated cell layer around placenta villi (Blond et al., 1999; Mi et al., 2000). Its 5′ UTR contains a LTR with a trophoblast-specific enhancer and a promoter region with a CpG island, whose methylation controls syncitin-1 expression (Matoušková et al., 2006; Gao et al., 2012). *HERV-W* also highlights how these elements can be important for pregnancy outcomes as it is hypomethylated and expressed in placenta, with lower expression in preeclampsia and IUGR placentas (Ruebner et al., 2013).

Placenta methylation at gene promoters is highly variable between individuals, with increased variability in term compared to first trimester placentas (Novakovic et al., 2011). However, there is also variability across a placenta depending on the sampling site (Janssen et al., 2015) which may reflect cellular composition.

Placental methylation at many genes has been associated with pregnancy outcomes. Placentas from pregnancies with preeclampsia have higher global DNA methylation and within this, blood pressure increases correlate with methylation increases (Kulkarni et al., 2011). Studies looking at preterm infants found lower SAM:SAH ratio and global methylation (Khot et al., 2017). Sinclair et al. (2007) found 15 loci where methylation associated with birthweight, of which four had corresponding changes in transcript levels in placenta.

In recent years, genome-wide DNA methylation values have been used to show discrepancies between chronological and biological age. Such “epigenetic clocks” use algorithms to calculate biological age on the basis of hundreds to thousands of CpG sites across the genome (Horvath, 2013). By calculating multi-organ clocks it is possible to predict clinically useful biomarkers associated with age-related disease and mortality (Lu et al., 2019). Whilst a pan-tissue epigenetic clock cannot reliably estimate gestational age, Lee et al. (2019) generated bioinformatic pipelines to generate placenta-derived epigenetic clocks that can track gestational age.

Deviations from normal placenta aging have been reported for several pregnancy complications. Accelerated epigenetic aging has been linked to early onset preeclampsia (Mayne et al., 2017), and lower fetal weight (Tekola-Ayele et al., 2019), while deceleration has been associated with maternal weight gain during pregnancy (Workalemahu et al., 2021). Together this indicates that both epigenetic age acceleration and deceleration are associated with distinct risk and protective factors, with studies from many laboratories are trying to identify distinct, tissue and cell-type specific trajectories to predict pregnancy outcomes.

### 3.3 Placenta-specific imprinting

Although the placenta is hypomethylated overall compared to somatic tissue, it exclusively maintains PoO methylation and expression at many transient gDMRs (Figure 2). In humans, over 150 maternally methylated DMRs have been identified in the placenta, around half of which have confirmed paternal expression (Yuen et al., 2011; Barbaux et al., 2012; Sanchez-Delgado et al., 2015; Hamada et al., 2016; Hanna et al., 2016; Sanchez-Delgado et al., 2016). Placenta-specific imprinting is highly polymorphic between individuals in humans and poorly conserved between humans and mice, though it may be more similar in other primates (Hanna et al., 2016; Sanchez-Delgado et al., 2016).

Hanna et al. (2016) found a monoallelic methylation in 50% of heterozygous samples at selected DMRs, looking just at trophoblast and villi cells to remove differences from cell composition, and there was significantly less variability at the same sites in somatic tissues. Complementary studies have showed that of 104 placenta-specific gDMRs studied, 52% intervals possess low methylation and biallelic expression in multiple term biopsies (Sanchez-Delgado et al., 2016; Monteagudo-Sanchez et al., 2019), although the frequency was variable between loci. It is unknown if this variation arises from the interactions with in cis genetic variants, environmental exposures, or is truly stochastic.

One well characterised transiently imprinted gene in mice is *Zdbf2* (zinc finger DBF-type containing 2) (Duffié et al., 2014). Generally, imprinting is poorly conserved between humans and mice, but the methylation pattern is the same at *Zdbf2*. The locus has a maternal pre-implantation gDMR, causing paternal expression of an alternative long isoform Liz (Duffié et al., 2014). This leads to creation of a paternal secondary DMR, which blocks the repressive H3K27me3 mark allowing paternal expression from the canonical *Zdbf2* promoter. In somatic tissue, the maternal gDMR is lost after implantation, leaving the paternal sDMR and *Zdbf2* expression. In extraembryonic lineages, including the placenta, the maternal gDMR and Liz expression are maintained instead (Kobayashi et al., 2009; Duffie et al., 2014). In humans, decreases in placental *ZDBF2* expression have been associated with intrauterine growth restriction (Monteagudo-Sánchez et al., 2019).

The impact of imprinted genes in placenta has been well studied in mice. Mouse models have demonstrated that generally deletions of genes which are paternally expressed, including *Igf2*, *Peg1* and *Peg3*, reduce placental size and increase the incidence of IUGR (DeChiara et al., 1991; Lefebvre et al., 1998). The opposite happens with deletions on maternally expressed genes, such as *Grb10* and *Phlda2*, causing fetal over-growth (Charalambous et al., 2003; Salas et al., 2004).

In humans, IUGR has been associated with placental expression of the imprinted *GPR1-AS1* and *ZDBF2* genes (Monteagudo-Sánchez et al., 2019). It is also associated with increases in *HYMA1* expression, and, in girls only, lower *PLAGL1* expression (Iglesias-Platas et al., 2014). *PLAGL1* is a transcription factor and its expression changes impact a network of genes downstream.

## 4 Placenta-specific effects of environment on health

The placenta is particularly interesting in the context of DOHaD because early environment can influence placental function, impacting development and pregnancy outcomes. Further, the placenta has a large impact on fetal development and placenta-related adverse pregnancy outcomes can influence later life disease. Placental function can be influenced by many environmental exposures, such as maternal smoking and obesity (Reijnders et al., 2019; Scott et al., 2022). Increased physical activity before and during early pregnancy can reduce the incidence of preeclampsia (Aune et al., 2014). ART is associated with increased risk of IUGR or premature birth (Camprubí et al., 2013).

Adequate nutrition in the first trimester and periconceptional folic acid supplementation are associated with lower resistance of the uterine and umbilical arteries in second and third trimesters, in which high resistance can be associated with preeclampsia and fetal growth restriction risk (Reijnders et al., 2019). The effect of the previously discussed Dutch Famine has also been studied, with famine exposure reducing placenta weight, birthweight and increasing preterm births, stillbirths and neonatal death (Susser and Stein, 1994; Lumey, 1998). A study in Gambia found that periconceptional multiple micronutrient supplementation improved placenta vascular function (Owens et al., 2015).

A systematic analysis of the effects of micronutrient supplementation looked at six placenta-related outcomes: preeclampsia, small for gestational age, low birthweight, preterm birth, stillbirth and maternal death (Kinshella et al., 2021). No single factor affected all six, but one or more were affected by vitamins C and E, vitamin D, vitamin D and calcium co-supplementation, calcium, iron and/or folic acid, zinc and multiple micronutrient supplementation. In another review, 14 of 25 nutritional factors reviewed were significantly associated with preeclampsia incidence, including vitamin D deficiency and high serum iron (Kinshella et al., 2022).

### 4.1 Placenta-specific effects of environment on methylation

In rodents, placenta methylation is affected by maternal diet. A gestational high-fat diet leads to placenta global hypomethylation and changes in methylation at enzymes involved in epigenetic machinery and expression (Gallou-Kabani et al., 2010). In mice, folate deficiency leads to lower overall placental methylation (Mahajan et al., 2019). There are also changes at many genes in the transcriptome and methylome, and the genes vary with sex, even being involved in different biological functions (Gabory et al., 2012).

Knowledge on the effect of diet on human placental methylation is limited, but changes in methylation linked to many other maternal exposures have been more widely studied. For example, ART has been linked to methylation changes in placenta, specifically reduced methylation at the *H19* and *MEST* DMRs with an increase in *H19* expression (Nelissen et al., 2013). In another study, ART placentas had increased expression of *INSIG1* and *SREBF1*, linked to cholesterol metabolism, with decreased methylation (Lou et al., 2014). A recent review looked at other exposures associated with methylation changes in placentas both globally and at specific sites, including air pollution, maternal smoking, bisphenol A and trace metals (Mortillo and Marsit, 2022). They observed that the effects are generally stronger when the exposure occurs in the first trimester. In other studies, maternal depression and anxiety have also been linked to a decrease in overall placenta methylation levels, with some changes in gene expression (Chen et al., 2014).

There are some studies on the effects of maternal diet on placental methylation in humans, for example, prenatal vitamin intake has been associated with a small reduction in placental methylation, including at sites associated with neuronal developmental pathways (Dou et al., 2022). Looking at specific components, folate has been widely studied during pregnancy, as its deficiency is associated with low birth weight, preterm birth, spontaneous abortion and birth outcomes including neural tube defects (Tamura and Picciano, 2006; Fekete et al., 2010; Greenberg et al., 2016; Hoffbrand et al., 2014). One-carbon components, including folate, have been studied in complicated pregnancies to see how they affect DNA methylation. There are three components important for folate transport through the placenta: Three main components participate in this process: folate receptor α (*FRα)*, reduced folate carrier (*RFC*) and proton-coupled folate transporter (*PCFT*) (Solanky et al., 2010; Castano-Morena et al., 2020). *FRα* mRNA was lower in preterm placentas compared to term, which correlated with increased methylation on the fetal side (chorionic plate). The increased methylation in preterm placentas also correlated with higher folate and lower B12 concentrations in the cord blood (Piñuñuri et al., 2020). The syncytiotrophoblasts in IUGR placentas had lower folate update and reduced levels of *RFC* but not *FR-α* (Chen et al., 2018).

In preterm placentas, expression of MAT2A and AHCY, enzymes involved in producing SAM, is higher in preterm placentas, and a SAH:SAM ratio, giving a lower methylation potential (Khot et al., 2017). These studies highlight how maternal nutrition can have an effect on methylation through one-carbon cycle components. However, not much is known still about how the overall methylation changes are acting to alter the function of the placenta.

## 5 Future directions

The placental epigenome is hypomethylated and hypervariable so has the potential for creating large inter-individual differences. This gives it great potential as a tissue to investigate the effects of environmental exposures on DNA methylation. So far, there has been limited study on the effects non-nutritional exposures and no study on the correlation in methylation at MEs between embryonic and extra-embryonic tissues. If MEs in placenta and somatic tissue are matched, this could suggest MEs are established before the trophectoderm lineage separates. Many of the MEs identified as responsive to season of conception in The Gambia overlap placenta DMRs which could suggest a mechanistic link, perhaps an issue with residual DMRs rather than somatically acquired. However, this is unlikely to explain all MEs, those that are not related to gDMRs must have a different mechanism of variability.

MEs are also known to disappear with age, so a readily obtainable early life tissue such as the placenta is an excellent place to look. This would also diversify the tissues used, as MEs are commonly identified only in cord blood. It is possible that more variation may be maintained in placenta as it is only needed for 9 months so is not regulated as highly as somatic tissue, or the unique epigenetic landscape may play an important role. Perhaps if there is a correlation, the placenta epigenome and transcriptome would serve as a good biomarker for later life disease. There is need for more work on MEs in humans to determine the effects of genetic variation and their relationship to environmental exposures and health.
